# Transient regulatory-T-cell interruption promotes skin-resident memory T cells mediated tumor protection

**DOI:** 10.1038/s41598-023-36884-w

**Published:** 2023-07-05

**Authors:** Shushu Zhao, Shuting Wu, Sheng Jiang, Xiaoyu Zhou, Gan Zhao, Bin Wang

**Affiliations:** 1 Key Laboratory of Medical Molecular Virology of the Ministry of Health and Ministry of Education, School of Basic Medical Sciences, Fudan University, Shanghai, 200032 China; 2 Shanghai Institute of Infectious Disease and Biosecurity, Fudan University, Shanghai, 200032 China; 3 National Clinical Research Center for Aging and Medicine, Huashan Hospital, Fudan University, Shanghai, 200032 China; 4 Present Address: Department of Genetics, Yale University School of Medicine, New Haven, CT 06520 USA; 5 Present Address: Advaccine Biopharmaceutics (Suzhou) Co. Ltd., Suzhou, 215000 China

**Keywords:** Cancer, Tumour immunology

## Abstract

Most cancer immunotherapy approaches aim to stimulate cytotoxic CD8^+^ T lymphocytes to reject tumor cells. Due to the tumor-mediated suppressive micro-environment, of which the major contributor is regulatory T cells (Tregs), promising preclinical approaches were disappointing in clinical settings. Our recent study demonstrated that transient interruption of Tregs could induce CD8^+^ T cell responses to reject tumors in an animal model. The long-term tumor protective effect has yet not to be investigated. In this study, mice with Treg depletion rejected tumors and were rechallenged to study anti-tumor memory immune responses. The effects of major immune cell subsets on tumor protection were explored. Finally, we demonstrate that transient depletion of Tregs during primary tumor challenge can result in long-lasting protection against the tumor rechallenge. Skin-resident memory T cells (sT_RM_) were major factors in rejecting rechallenged tumors even when peripheral T cells were deficient. These findings highlight a promising strategy for empowering tissue-resident memory T cells for cancer prevention and immunotherapy in humans by interrupting Tregs.

## Introduction

Cancer-targeted treatments have been the focus of research for several decades. According to the cancer immunoediting theory, during the early stage of tumor development, the immune system's innate and adaptive responses coordinate to protect the host through immunosurveillance. However, some tumor cells can escape immune elimination. One of the mechanisms by which this occurs is the assistance of immunosuppressive regulatory T cells (Tregs)^1^. Therefore, modulation of Tregs has emerged as a promising approach in cancer therapy. Tregs depletion using anti-CD25 or anti-CTLA4 monoclonal antibodies has shown positive effects in pre-clinical studies^2^ or clinical application^3^. This approach represses Tregs and enhances effector T cell (Teff) activation. In our previous study, we found that transient depletion of Tregs in tumor-draining lymph nodes (TDLN) by a surgical ligation during a short time window effectively activated CD8^+^T cells and inhibited tumor growth^4^. However, many questions remained unanswered, such as whether hosts that have rejected tumors can defend against rechallenge homogeneous tumors effectively, whether metastatic tumors rebound after Tregs recovery at normal level, and whether the specific anti-tumor responses elicited can generate memory T responses.

In the process of solid tumor elimination, the endothelial cells and keratinocytes increase the expression of chemokines such as ICAM-1, CXCL9, and CXCL10, attracting Teff into the tumor's adjacent inflamed skin^5^. As for the T cells of long-term residents in the skin (termed skin resident T cells or sT_RM_), the best characterization is the expression of CD69 and CD103. Expression of CD69 on T-cell indicates its activation and will reduce the level of S1P receptor correspondingly on this cell, resulting in sT_RM_ retention in tissues, such as the skin. CD69 promotes the early lodgment of sT_RM_ in the skin, whereas the expression of CD103 is induced later, which binds to E-cadherin to maintain their persistence in that location^6^. It has been reported that following local antigen encounters, sT_RM_ responded quickly and strongly both in virus re-infection and cancer rechallenge^7,8^. In addition, sT_RM_ recruits circulating memory T cells and triggers the spread of cytotoxic CD8^+^T cell responses^6,9^.

Here, we demonstrate that Tregs transient depletion during primary antigen encounter amplifies anti-tumor immunity against homogeneous tumor rechallenge. Transient depletion of Tregs promotes the formation of sT_RM_ cells, which provide long-term tumor protection. Even when peripheral CD8^+^T cell are depleted, sT_RM_ cells can suppress tumor development. This work has generally shed light on potentiating the current immunotherapeutic protocol for human cancer treatment.

## Results

### Tregs transient depletion induced long-term tumor rejection responses

**Figure 1:**
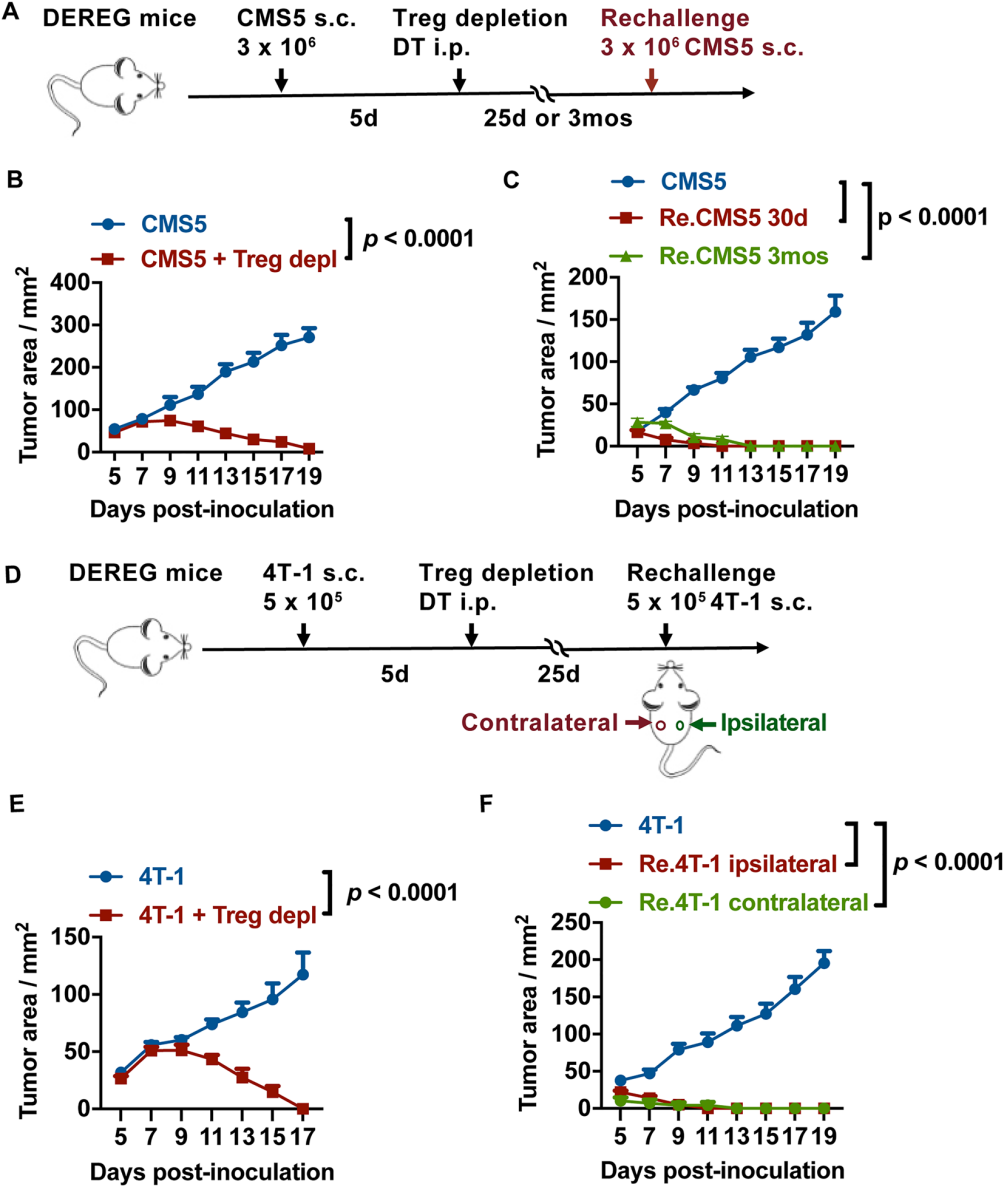
Treg depletion induced long-term tumor rejection responses. (**A**) Schematic illustration of the rechallenged CMS5 tumor rejection model. DEREG mice were inoculated s.c. with CMS5 tumor cells. DT was injected i.p. to delete Tregs. After 25 days or 3 months, DEREG mice were rechallenged with CMS5. (**B**) Tumor development after CMS5 primary challenge (n ≥ 8). (**C**) Tumor development after CMS5 rechallenge (n ≥ 8). (**D**) Schematic illustration of the rechallenged 4T-1 tumor rejection model. The 4 T-1 tumor was challenged in this protocol. In the rechallenge process, tumors were inoculated s.c. on the right flank (Re.4T-1 ipsilateral) or the left flank (Re.4T-1 contralateral). (**E**) Tumor growth after 4T-1 challenge (n = 8). (**F**) Tumor growth after 4T-1 rechallenge (n = 8). Two-way ANOVA followed by multiple comparisons for tumor growth curve analysis was used. Error bars represent mean $\pm$ SEM.

In our previous study, we found Treg depletion at the early stage of tumor development induced strong anti-tumor responses and resulted in tumor eradication^4^. To further investigate the tumor protection effects, we conducted a similar study with an additional challenge at later time points with the homologous tumor cells to determine if the anti-tumor activity could be sustained. DEREG (Foxp3-DTR-EGFP) mice were inoculated with CMS5 fibrosarcoma tumor cells, and Tregs were depleted by injecting diphtheria toxin (DT). After DT injection, Tregs were entirely depleted within two days but returned to normal levels after 10 days (supplementary Fig. S1A). To explore long-term anti-tumor protection, mice were rechallenged with CMS5 tumor cells 30 days or 3 months post the initial challenge (Fig. 1A). CMS5 tumors were rejected in Treg-knockout DEREG mice during the first tumor challenge, whereas CMS5 tumors in the control littermate wild type mice developed aggressively (Fig. 1B). After tumor rechallenge, CMS5 tumors were rejected rapidly and completely in both the short-term and long-term groups (Fig. 1C). To study the tumor protection effect without Treg interruption, tumor excision was also performed. CMS5 tumor excision failed to provide complete protection against tumor rechallenge (supplementary Fig. S1B), suggesting that Treg depletion stimulated strong tumor rejection responses. Additionally, a 4T-1 tumor model was established to verify this process. To investigate the influence of tumor rechallenge position, mice were rechallenged either in the ipsilateral or contralateral position of the first tumor inoculation site (Fig. 1D). The results were consistent with the CMS5 model study. After Treg depletion for the first challenge, 4T-1 tumors were rejected (Fig. 1E) and tumors were completely repelled after rechallenge regardless of the tumor injection position (Fig. 1F). These results collectively demonstrate that the long-term tumor rejection responses was mediated by Treg transient depletion.

### T cell responses promoted rechallenged tumor rejection

**Figure 2:**
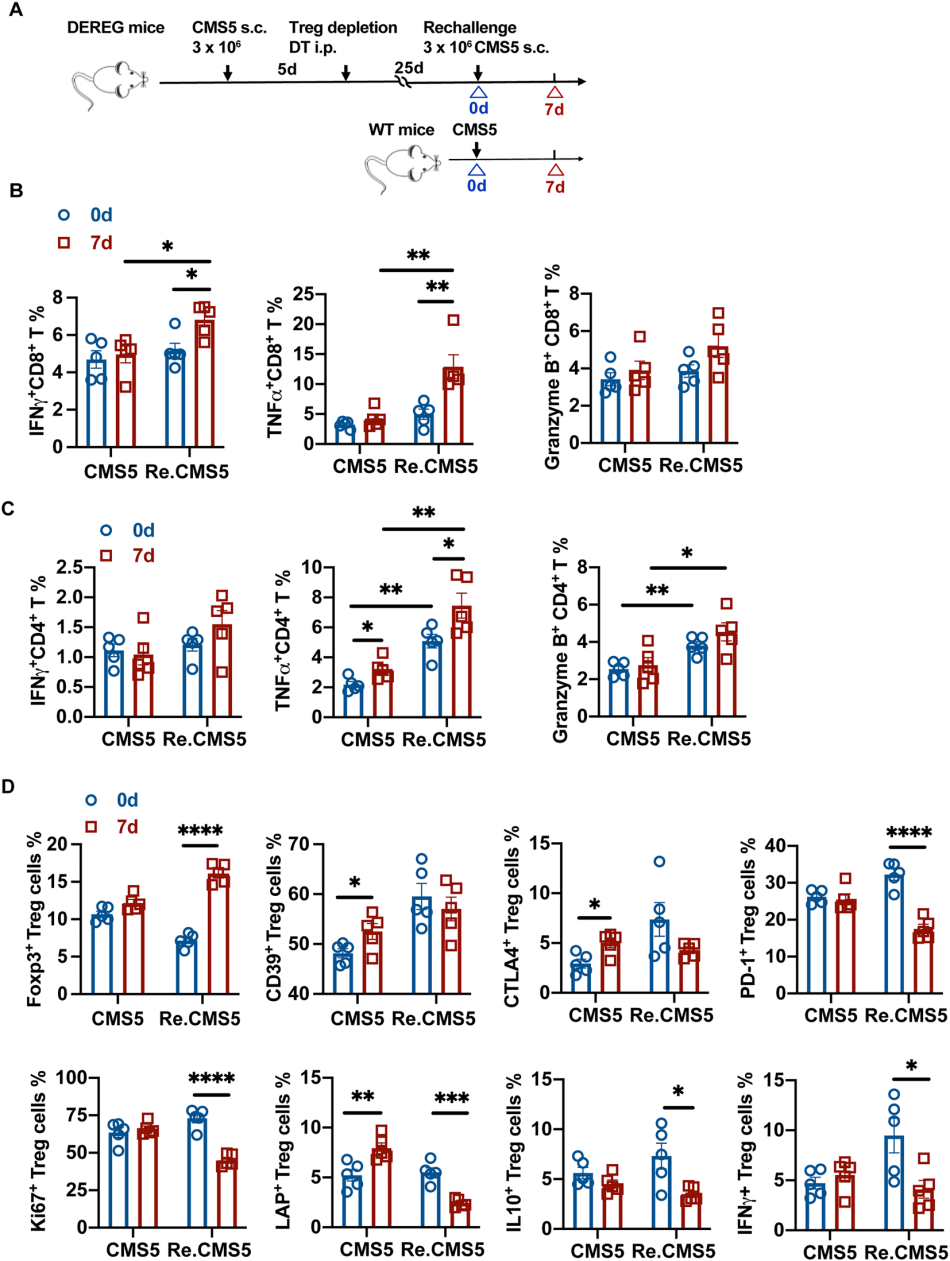
T cell responses promoted rechallenged tumor rejection. (**A**) Experimental scheme of T cells responses analysis at the indicated time points. Samples were acquired the day before and 7 days post CMS5 rechallenge (Re.CMS5). Samples collected from littermate wild type (WT) mice with no treatment and CMS5 bearing for 7 days were as control (CMS5). (**B**) Splenic CD4^+^ T cells expression of IFN$\gamma$, TNF$\alpha$ and granzyme B (n = 5). (**C**) Splenic CD8^+^ T cells expression of IFNγ, TNFα and granzyme B (n = 5). (**D**) Expression of CD39, CTLA4, PD-1, Ki67, LAP, IL-10 and IFNγ by Tregs from tumor draining lymph nodes (n = 5). An unpaired Student’s t-test was used. ****p < 0.0001, ***p < 0.001, **p < 0.01, *p < 0.05. Error bars represent mean $\pm$ SEM. See also Fig. S2.

To determine effector T cells (Teff) involvement in rechallenged tumor eliminations, we examined Teff cells responses specific to CMS5 tumor cells (Fig. 2A). The secretion of IFN$\gamma$,TNF$\alpha ,$ and granzyme B by splenic CD8^+^ and CD4^+^ T cells were analyzed. Both the gating strategy and PMA and ionomycin stimulation controls were shown in supplementary Fig. S2A. After the CMS5 tumor rechallenge, CD8^+^ T cells produced more IFNγ and TNFα than those before the tumor rechallenge or in the CMS5 inoculated control group (Fig. 2B, supplementary Fig. S2B). CD4^+^ T cells from the rechallenged mice secreted more TNFα$,$ and granzyme B than in CMS5 inoculated control. Tumor rechallenge significantly stimulated CD4^+^ T cells expression of TNFα (Fig. 2C). We also assessed the level of serum cytokines during the primary and rechallenged tumor rejective responses (supplementary Fig. S2C). Consistent with the preceding findings, IFNγ secretion was increased both in the primary and the rechallenged tumor rejective responses, and serum TNFα level was increased significantly during tumor rechallenge (supplementary Fig. S2D). These results suggested that CMS5 rechallenge stimulated peripheral cytotoxic T cells responses to repel tumor growth.

The suppressive function of Tregs was further explored to reveal their effects after tumor rechallenge. Supplementary Fig. S2E depicts the gating strategy for analyzing the function of Treg cells. There was no discernible difference in Treg cell percentage between CMS5 7 days and 0 day (Naïve group). However, Tregs increased significantly after CMS5 tumor rechallenge. Next, we explored whether these increased Tregs would contribute to immune suppression. In CMS5 inoculated control, Tregs increased the expressions of CD39, CTLA4, and latency-associated protein (LAP) 7 days post tumor challenge, suggesting the enhanced suppressive function of Tregs. Interestingly, although the changes of CD39 and CTLA-4 expression were not statistically significant, the expression of PD-1 and Ki67 by Tregs were reduced significantly, and lower levels of IL-10, LAP and IFNγ were produced following CMS5 rechallenge (Fig. 2D, supplementary Fig. S2F). These results indicate that Treg suppressive function was inhibited during tumor rechallenge.

### Rechallenged tumors were rejected even with circulating immune cells depletion

**Figure 3:**
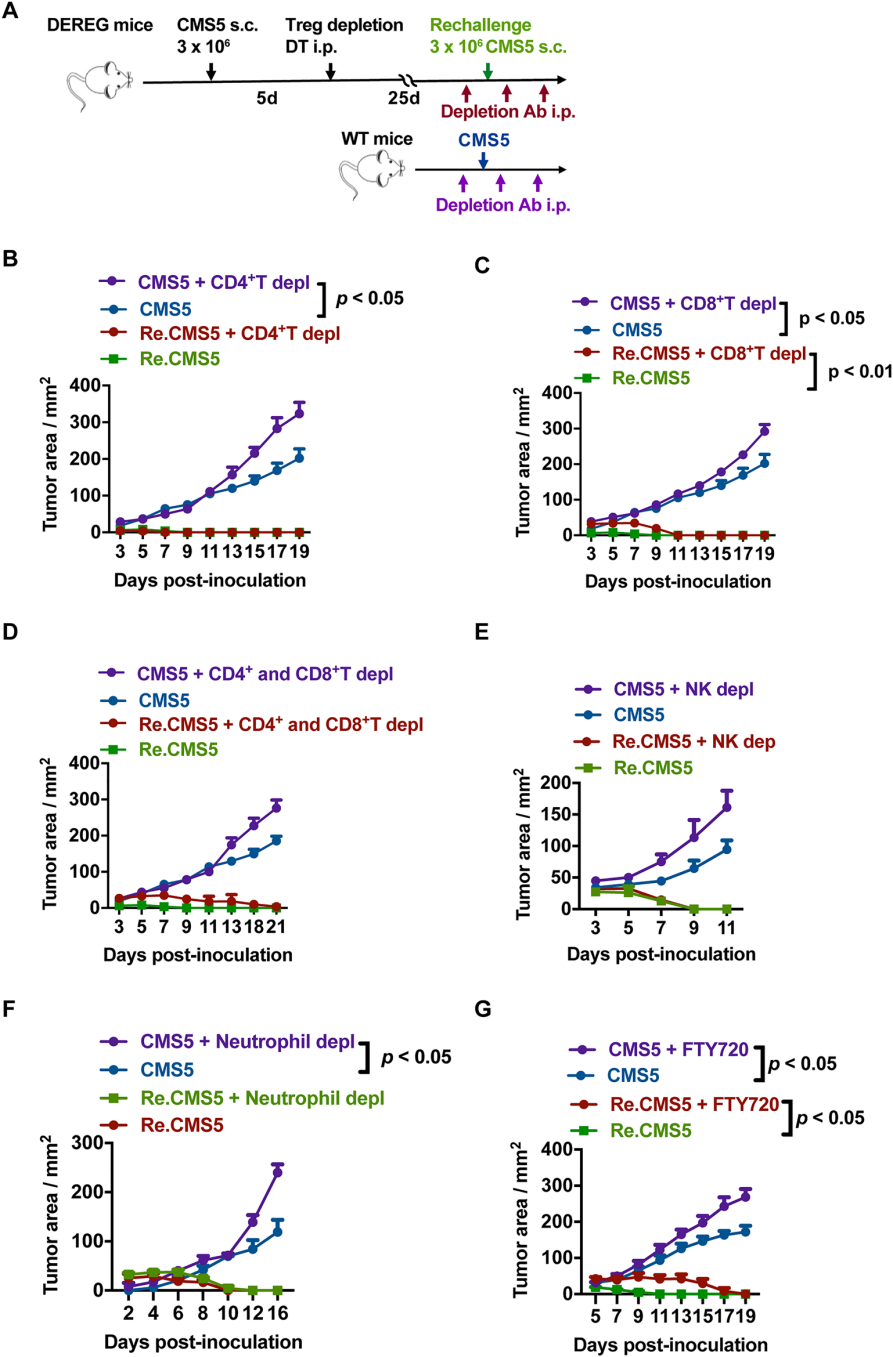
The rechallenged tumors were rejected even with circulating immune cell depletion. (**A**) Schematic illustration of immune cells depletion with antibodies during tumor rechallenge. (**B**) Tumor development with CD4^+^ T cells depletion (n = 5). (**C**) Tumor development with CD8^+^ T cells depletion (n = 5). (**D**) Tumor development with the depletion of CD4^+^ T and CD8^+^ T cells (n = 5). (**E**) Tumor growth with NK cell depletion (n = 5). (**F**) Tumor growth with neutrophil depletion (n = 5). (**G**) Tumor development with FTY720 treatment (n = 5). Two-way ANOVA followed by multiple comparisons for tumor growth curve analysis was used. Error bars represent mean $\pm$ SEM. See also Fig. S3.

To explore whether T cells were the crucial factors in rechallenged anti-tumor responses, we depleted peripheral T cells during tumor rechallenge and tracked the tumor development (Fig. 3A). Through analyzing T cells in PBMCs by flow cytometry, we found that majority of CD4 + or CD8 + T cells were removed by injecting anti-CD4 or anti-CD8 antibody (supplementary Fig. S3A). Moreover, CMS5 tumors proceeded more aggressively when lacking CD4^+^ T or CD8^+^ T cells in the control groups (Fig. 3B–D), suggesting that CD4^+^, CD8^+^, or both T cells are essential in suppressing tumor growth. Nevertheless, even with CD4^+^ T cell depletion, tumors were quickly eradicated in the groups rechallenged with CMS5 (Fig. 3B). Rechallenged tumors were finally repelled after a period of struggle in the CD8^+^T cell or both CD4^+^T and CD8^+^T cell depletion groups (Fig. 3C–D). None of the groups in the rechallenge process had a the same tumor burden as seen in the CMS5 inoculated control groups. These results indicate that peripheral CD4^+^ T and CD8^+^ T cells responded to the rechallenged tumors but played no role in tumor rejections. Some other factors must mediate tumor eradication when CD4^+^ T and CD8^+^ T cells were absent.

To further explore what immune cell subsets worked to eradicate rechallenged tumor cells, we studied the necessity of several other immune surveillance-related cells during tumor rechallenge. Natural killer cells (NKs) and neutrophils were reported to be essential in suppressing tumor development. Therefore, NKs or neutrophils were depleted by anti-asialo GM1 or anti-Ly6G antibodies during tumor rechallenge (supplementary Fig. S3B). Although tumors grew faster with NKs or neutrophils depletion in control groups, rechallenged tumors were rejected with or without either of these immune cells (Fig. 3E,F). FTY720, an immunomodulating drug that acts as a sphingosine 1-phosphate (S1P) antagonist, was then used to sequester lymphocytes in lymph nodes. After FTY720 treatment, significantly fewer immune cells were detected in peripheral blood (supplementary Fig. S3C). We found that rechallenged tumor cells survived longer in the mice with FTY720 treatments but eventually eradicated completely (Fig. 3G). As this result was similar to tumor growth after both CD4^+^T and CD8^+^T depletion with antibodies (Fig. 3D), it provides solid evidence that rechallenged tumors clearance can be independent of peripheral T cells.

### sT_RM_ cells expanded after tumor rechallenge

**Figure 4:**
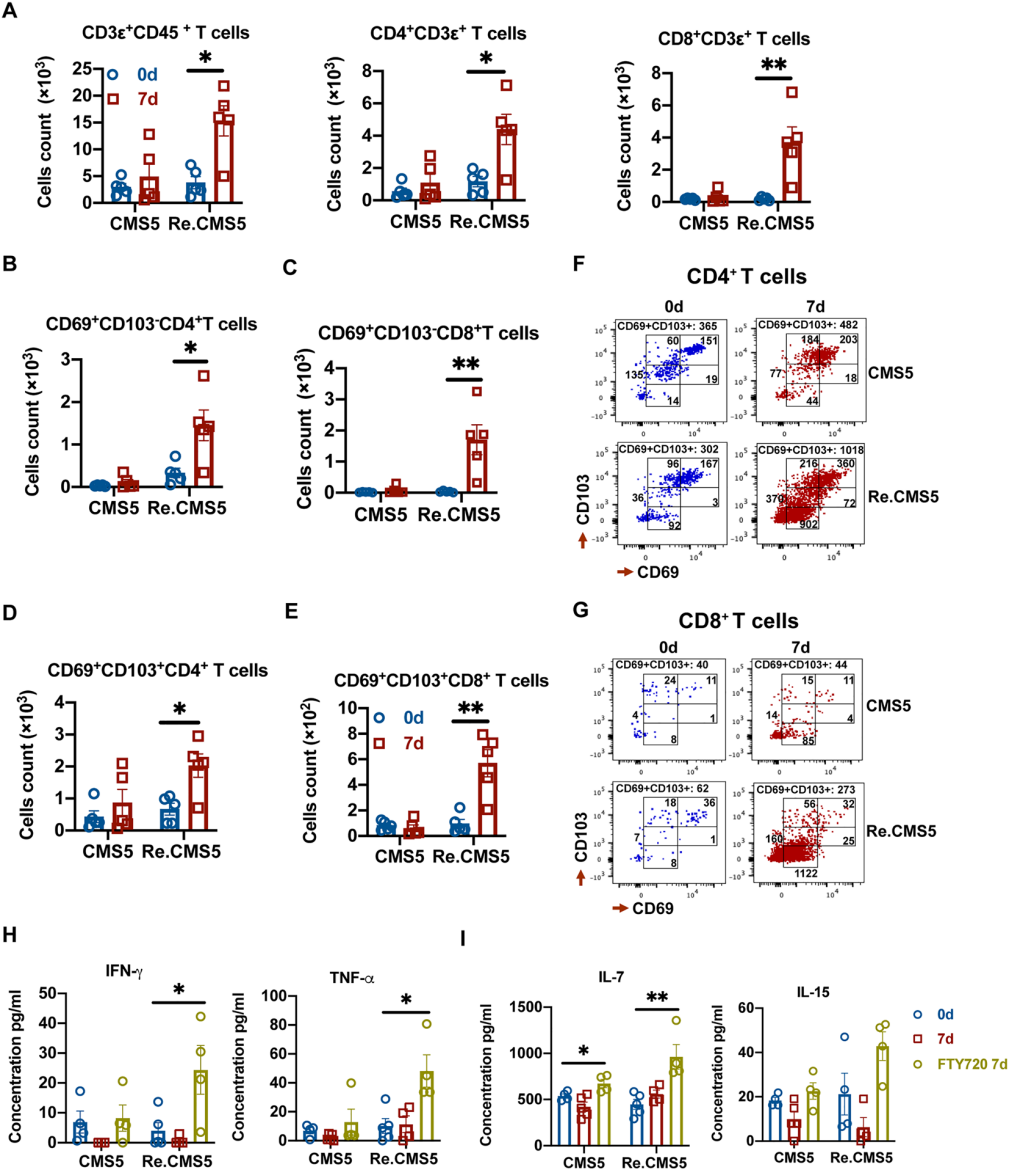
sT_RM_ cells expanded after tumor rechallenge. (**A**) T cells infiltrate the skin after tumor rechallenge (n = 5). (**B-E)** The number of CD69^+^CD103^−^ activated T cells and CD69^+^CD103^+^ T_RM_ cells infiltrated in the skin (n = 5). (**F-G**) The representative results of skin infiltrating CD4^+^ and CD8^+^ T cells analysis by flow cytometry. (**H-I**) ELISA analysis of cytokines level in skin macroenvironment. An unpaired Student’s t-test was used. **p < 0.01, *p < 0.05. Error bars represent mean $\pm$ SEM. See also Fig. S4.

Given the rechallenged tumor rejection without peripheral T cell involvement, we hypothesized that tissues resident T cells, especially sT_RM_ cells, could mediate long-term protective responses against tumors. To test this hypothesis, skin infiltrating T cells were analyzed by flow cytometry after the CMS5 rechallenge. The number of CD3^+^, CD4^+^, and CD8^+^ T cells infiltrated in the skin increased significantly after the CMS5 rechallenge. At the same time, T cell expansions in the skin of CMS5 inoculated control groups showed no change (Fig. 4A). Moreover, increased activated peripheral CD4^+^ and CD8^+^ CD69^+^CD103^-^T cells were recruited to skin following CMS5 rechallenge (Fig. 4B,C). Most importantly, resident CD4^+^ and CD8^+^ CD69^+^CD103^+^ T_RM_ subsets expanded significantly after the CMS5 rechallenge (Fig. 4D,E). Figure 4F,G depicts the representative flow cytometry results.

To exclude infiltration of peripheral T cells to skin and measure skin resident T cells’ expansion, mice were treated with FTY720 during CMS5 rechallenge. After FTY720 treatment, skin T cells detected decreased obviously 7 days post tumor rechallenge (supplementary Fig. S2A). However, no significant reduction was observed in CD4^+^ and CD8^+^ CD69^+^CD103^+^ sT_RM_ cells on 7 days with FTY720 treatment versus no treatment. However, CD4^+^ and CD8^+^ CD69^+^CD103^+^ sT_RM_ cells expanded with FTY720 treatment compared with 0 day group before the tumor rechallenge (supplementary Fig. S4B). The cytokine expressions in the areas of skin where the tumor rechallenged were analyzed by ELISA. Interestingly, there were obviously no differences at concentrations of IFNγ, TNFα, IL-7, and IL-15 from CMS5 rechallenged groups at 0 day versus 7 days. Nevertheless, all tested cytokines level increased dramatically after FTY720 treatment, as FTY720 limited migration of resident T cells allowing cytokines to be released in situ (Fig. 4H,I). Collectively, these data demonstrate that CMS5 rechallenge stimulates the expansion of sT_RM_ and promotes the recruitment of T cells into the skin with increased long-term survival by elevating expressions of IL-7 and IL-15 in the skin and enhancing the secretion of inflammatory cytokines IFNγ and TNFα to repel tumor.

### More T_RM_ generated in the skin after Treg interruption

**Figure 5:**
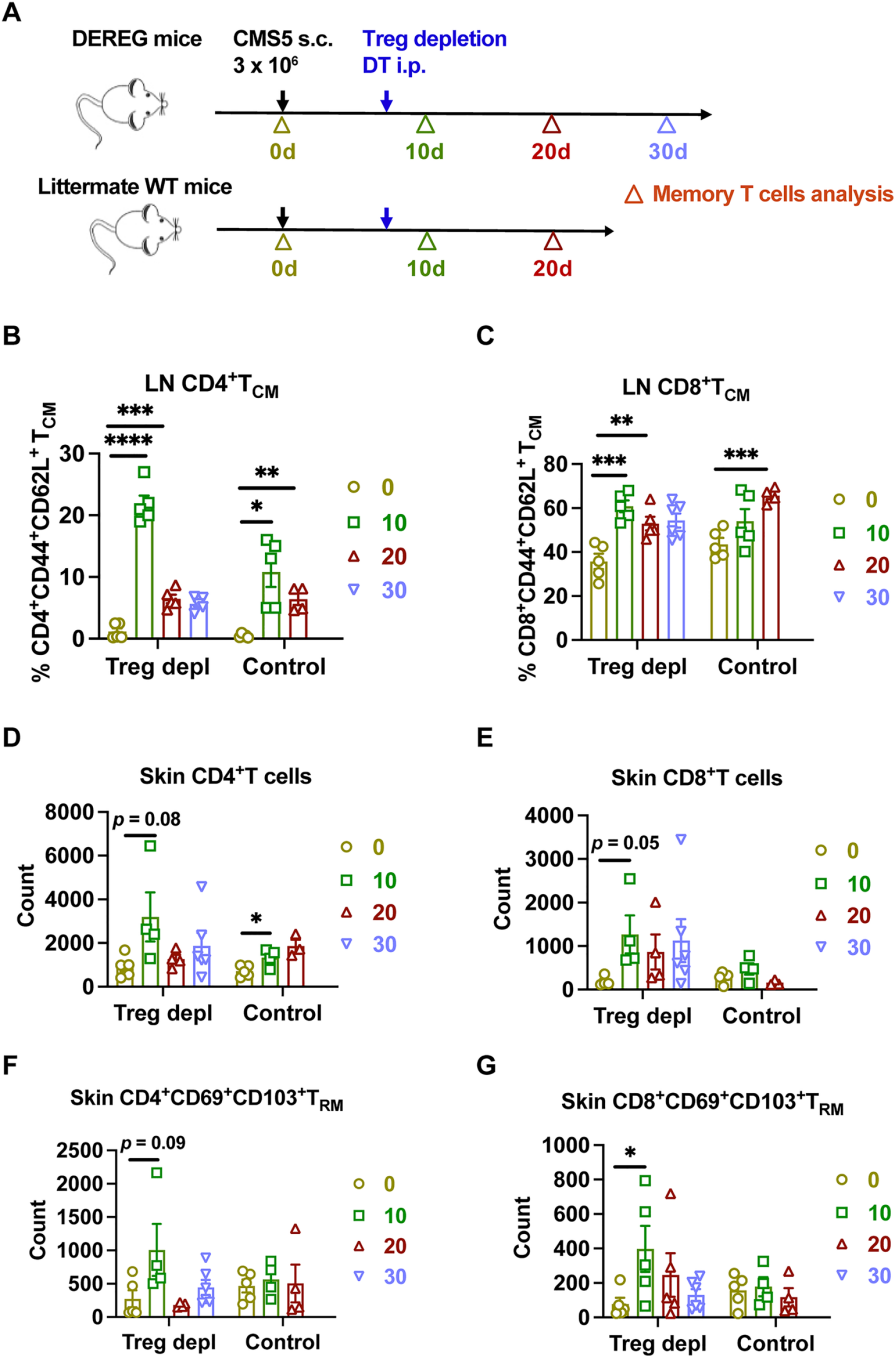
More T_RM_ generated in the skin after Treg interruption. (**A**) Experimental scheme of memory T cells analysis at the indicated time points during primary tumor challenge. (**B–C**) Analysis of CD4^+^ and CD8^+^ T_CM_ cell formation in tumor-draining lymph node (n = 5). (**D–E**) Skin infiltration of CD4^+^ and CD8^+^ T cells during primary tumor challenge (n = 5). (**F–G**) Analysis of CD4^+^ and CD8^+^ T_RM_ cell formation in the skin (n = 5). An unpaired Student’s t-test was used. ****p < 0.0001, ***p < 0.001, **p < 0.01, *p < 0.05. Error bars represent mean ± SEM.

To investigate how Treg interruption or tumor implantation influences the formation of memory T cells, we analyzed the dynamic changes of memory T cells in lymph node (LN), and tumor-adjacent skin after primary tumor challenge (Fig. 5A). Central memory T cells (T_CM_) are antigen-specific memory T cells that recirculate in secondary lymphoid organs including lymph nodes. Increased CD4^+^ and CD8^+^ T_CM_ were detected both in the Treg depletion group and control group after the tumor challenge (Fig. 5B,C). The percentages of CD4^+^ and CD8^+^ T_CM_ peaked on day 10 and shrank on day 20, except for CD8^+^ T_CM_ in the control group. Compared with day 0, more T_CM_ was generated in the tumor-draining lymph node on day 30 in the Treg depletion group and day 20 in the control group. Although counts of skin infiltrating CD4^+^ and CD8^+^ T cells increased, no statistical significance was found post-tumor injection in the Treg depletion group (Fig. 5D,E). A slightly increased number of CD4^+^ T cells was detected in the control group. By analyzing the generation of skin T_RM_, the number of CD4^+^ T_RM_ showed no significant change but with a mild increase on day 10 in the Treg depletion group (Fig. 5F). Much more CD8^+^ T_RM_ were observed in the skin on day 10 post-tumor challenge (Fig. 5G), but no significant difference was found in the control group. These results suggest that primary tumor challenge may promote the formation of T_CM_ in lymph nodes but has no impact on the generation of T_RM_. However, Treg disruption induces the generation of skin CD8^+^ T_RM_ which expand dramatically after the tumor rechallenge. This finding is further supported by our previous study, which observed that Treg cells trapped CD8^+^T cells in the tumor-draining lymph node, but the Treg depletion could unleash CD8^+^T cells egressed into tumor^4^. Finally, these released CD8^+^T cells form more T_RM_ in the skin to provide long-term protection against tumor growth.

### sT_RM_ deficiency resulted in rechallenged tumor progression

**Figure 6:**
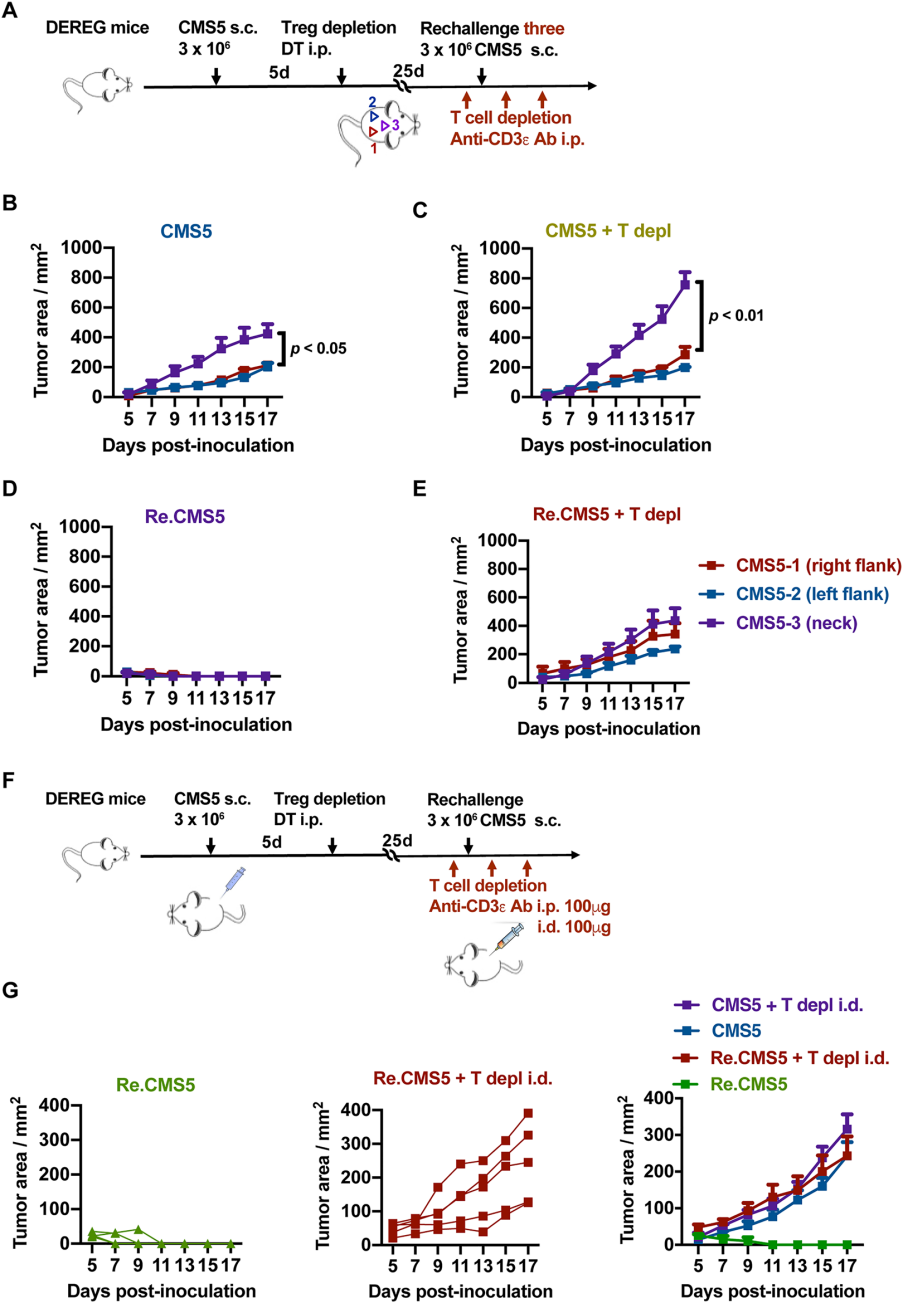
sT_RM_ deficiency resulted in rechallenged tumor progression. (**A**) Experimental description of multiple tumors inoculation in the absence of peripheral T cells. Briefly, the DEREG mice that have rejected CMS5 tumors by Tregs depletion were injected i.p. with 200 μg anti-CD3ε F(ab′)2 fragment antibodies once a week. Mice were rechallenged with CMS5 tumors in the right, left flank, and neck of the back, respectively (CMS5-1, CMS5-2, and CMS5-3). The littermate WT mice injected with T cells depletion antibodies and challenged with multiple tumors were as control. Tumor growth was monitored (**B–E**) (n = 5). (**F**) Illustration of an experimental procedure to observe the rechallenged CMS5 tumors development with sT_RM_ cell depletion. Tumor cells were injected in the right flank. And anti-CD3ε F(ab′)2 fragment antibodies were injected 100 μg i.p. and 100 μg i.d. in the right flank weekly. (**G**) Tumor growth was monitored (n = 5). Two-way ANOVA followed by multiple comparisons for tumor growth curve analysis was used. Error bars represent mean ± SEM.

To provide evidence that the effect of tumor rejection during rechallenge was mediated by sT_RM_ cells in vivo, we designed two approaches to cause sT_RM_ cell insufficiency to control rechallenged tumor progression. One was implanting more tumors in the tumor rechallenge protocol. DEREG mice were rechallenged s.c. with triple doses of CMS5 cells on three positions after rejecting primary CMS5 tumors by Tregs depletion (Fig. 6A). To rule out the influence of peripheral T cells, T cells were depleted using anti-CD3 $\varepsilon$ F(ab′)2 fragment antibodies once a week. Tumor development was monitored. All tumors grew rapidly in the CMS5 control group (Fig. 6B) and in the T cells depletion group (Fig. 6C). No tumor formation was observed in mice rechallenged with CMS5 (Fig. 6D). However, with peripheral T depletion, all tumors progressed smoothly after tumor rechallenge (Fig. 6E). This result suggests that sT_RM_ cells were insufficient to withstand the rechallenge with triple doses of tumor cells without peripheral T cells assistance. However, single-dose CMS5 tumor cells rechallenge failed to form solid tumors even with peripheral T cell depletion (supplementary Fig. S5A–B). The efficiency of T cells depletion was analyzed. The result showed that peripheral T cells were obliterated (supplementary Fig. S5C).

Enlightened by Park et al.^10^, who depleted sT_RM_ cells by intradermal (i.d.) injection of antibodies, we attempted to deplete resident T cells by injecting anti-CD3ε F(ab′)2 fragment antibodies intradermally. Peripheral T cells were also removed through i.p. injection of antibodies before tumor rechallenge (Fig. 6F). Through detecting T cells in the skin, lung, lymph node, and spleen, T cells were significantly decreased with anti-CD3 antibodies injection i.p. 200 μg or both i.p. and i.d. but 100 μg (supplementary Fig. S5D). However, when compared to i.p. single injection of antibodies, i.p. combined with i.d. injection of antibodies depleted more skin T cells (supplementary Fig. S5E). Tumor development was then monitored. Rechallenged CMS5 tumors progressed normally similar to the first inoculated CMS5 control tumors with antibodies i.d. injection (Fig. 6G). Taken together, these results show that sT_RM_ cells can potentially inhibit tumor development in the absence of peripheral T cells, and that rechallenged tumors developed normally when sT_RM_ cells are insufficient or incompetent.

## Discussion

In this study, we demonstrated that primary tumor development was interrupted and rejected by transient depletion of Tregs, and such an anti-tumor response could elicit long-term anti-tumor protection and prevent subsequent tumor rechallenge in the same host. Increased cytotoxic T cells and suppressed function of Tregs were observed during secondary anti-tumor responses. Interestingly, we found that rechallenged tumors were rejected even when peripheral T cells were depleted by antibody administrations and expected that other subsets of immune cells should contribute to the rejection and need to explore further.

The anti-tumor effects of NK cells may also contribute to tumor regression. Immunosurveillance of NK cells has been particularly prominent in the fibrosarcoma model, and IL-12 has been found to enhance the tumor-killing function of NK cells^11^. A study also revealed that NK cells could control the growth of fibrosarcoma by secreting IFN-$\gamma$ and inhibiting the transformation of neutrophils into immunosuppressed phenotype^12^. Moreover, it was shown in our experiment that the growth of CMS5 tumors inoculated in wild-type control mice was significantly accelerated after NK cells depletion (Fig. 3E), suggesting that NK cells did play some role in inhibiting the growth of CMS5 fibrosarcoma. However, antibody-mediated knockout NK cells failed to confirm their anti-tumor contribution in the CMS5 rechallenge group, suggesting the role of NK cells is limited.

The most intriguing results are the similar rechallenged tumors growth curve of the T cells depletion group with anti-CD4 antibody and anti-CD8 antibody treatments (Fig. 3B) in the FTY720 migration-blocking group (Fig. 3G). The rechallenged tumors could not grow out but kept in minimal size, suggesting that both T cells depletion antibodies and FTY720 blockade only affect peripheral T cells but failed to influence the function of T_RM_ cells.

The early discovery of T_RM_ cells was mostly related to anti-infection. T_RM_ cells in local tissues can rapidly initiate innate and adaptive immunities to establish a solid protective barrier against viral re-infection^13^. Other studies on T_RM_ cells in the tumor model had mainly focused on melanoma. CD103^+^TILs were later found to have more substantial effects on controlling tumor growth than the CD103^-^TILs in lung, colorectal, breast, and other tumors^14–16^. Inspired by these findings, some researchers promoted to induce T_RM_ cells through local vaccinations, including intradermal or nasal mucosal administrations, to achieve the prevention against tumor growth^17–19^. These studies have also found that blocking the peripheral immune cells by FTY720 or depleting T cells with antibodies did not affect T_RM_ cells and their anti-tumor function, which agrees with our current findings. Furthermore, we observed a twofold increase in the number of CD4^+^T_RM_ cells and a sixfold increase in the number of CD8^+^T_RM_ cells in the skin at the tumor rechallenge sites compared to the concurrent accumulation of many activated CD69^+^CD103^-^T cells into the skin prior to the rechallenge (Fig. 4F,G). Previous studies have found that skin T_RM_ cells could sustain and amplify their anti-tumor response through dendritic cells and facilitate their migrations into draining lymph nodes for spreading out of loaded antigens^20,21^. We further observed that sT_RM_ cells were proliferated (Fig. S4B) and secreted large amounts of pro-inflammatory factors into the skin environment to inhibit tumor growth after the FTY720 blockage of infiltrating activated CD69^+^CD103^-^T_RM_ cells into skin tissue (Fig. 4H). Although this result suggests that sT_RM_ cells could be functional and self-renewable after encountering tumor challenge, their ability to reject tumor rechallenge can be limited when sT_RM_ cells are confronted with significant tumor burdens (9 × 10^6^ tumor cells challenge versus 3 × 10^6^) or interrupted their function by intradermal injection of antibodies (Fig. 6).

A previous report had documented that skin could become vitiligo after tumors were surgically removed when CD4^+^T cells, including CD4^+^Treg cells, were depleted with antibodies in a melanoma model. A large number of memory CD8^+^T cells were observed in vitiligo sites to permanently protect the host against melanoma^22^. Further studies with tissues from melanoma patients also found the persistence of resident memory CD8^+^T cells for several years^23,24^. However, some studies reported that the formation and survival of T_RM_ cells in the epidermis needs TGF-β^25–27^, and inter-clonal competition for active TGF-β results in the replacement of bystander T_RM_ cells by antigen-specific T_RM_ cells^28^. This may explain why the number of CD69^+^CD103^+^ T_RM_ cells in the skin of mice showed no statistical difference between the naïve groups (CMS5 0d) and primary tumor rejected groups (Re.CMS5 0d, Fig. 4C,E). Another interesting finding in our study was that CMS5 tumor cells rechallenged at different locations on the back of an animal were all rejected (Fig. 6A), suggesting that sT_RM_ cells might be able to migrate freely within the skin. This phenomenon is supported by the previous finding in a skin infection model with HSV (herpes simplex virus) where fluorescence-labeled sT_RM_ cells patrolled between keratinocytes in dendritic behavior and were ready to find and eliminate pathogens^29^.

In summary, we demonstrate that transient depletion of Tregs during primary tumor challenge could lead to long-term tumor protection and induce sT_RM_ cells. This finding provides significant insight for Tregs targeting tumor immunotherapy and supports T_RM_ cells as a further target to improve immune-therapeutic responsiveness.

## Methods

### Mice

BALB/c mice (6–8 weeks of age, female) were purchased from Shanghai JieSiJie Laboratory Animal Co., Ltd. Foxp3-DTR-EGFP (DEREG) mice in C57BL/6 background were purchased from The Jackson Laboratory, USA, and maintained in our college animal facility and bred in Beijing HFK Bio-Technology Co., Ltd. DEREG mice in C57BL/6 background were backcrossed with BALB/c mice for more than 20 generations to get DEREG mice in BALB/c background. All animal experiments were approved by the Experimental Animal Ethics Committee of Shanghai Medical College (reference number: 20160225-115). All experiments were conducted in accordance with relevant guidelines and regulations, including the Animal Research: Reporting of In Vivo Experiments (ARRIVE) guidelines.

### Tumor cells lines

CMS5 and 4T-1 tumor cells were kindly provided by Dr. Zhenzhou Wu (Nankai University, Tianjin, China) and Dr. Minghui Zhang (Tsinghua University, Beijing, China). Both tumor cells were cultured in RPMI 1640 (BI) supplemented with 10% FBS (BI), 100 U/mL penicillin, and 100 mg/mL streptomycin at 37 °C 5% CO2.

### Tumor inoculation and measurement

For subcutaneous inoculation, 3 × 10^6^ CMS5 or 5 × 10^5^ 4T-1 tumor cells suspended in 100 ml PBS were injected into the right flank. Developing tumors were monitored every other day using a digital caliper, and tumor size was calculated as tumor length × width.

### Tumor specific Teff cells responses analysis

To analyze antigen-specific T responses, mice were sacrificed, and spleens were collected at the indicated time points. Spleens were cut into pieces and ground into single-cell suspension. Red-cell lysis buffer was used to get rid of red blood cells. Then the splenic cells were restimulated with tumor cells in vitro for 48 h. Briefly, CMS5 cells were plated in 96-well round bottom plates, with 2000 cells in each well. The single cell suspensions of samples were added with around 5 × 10^5^ cells per sample. To sustain lymphocyte viability, the culture medium was RPMI 1640 complete medium containing 0.5 μg/ml anti-CD28 (eBioscience) and 0.2 ug/ml recombinant mouse IL-2 (PeproTech). To detect intracellular cytokines, brefeldin A (eBioscience) was added 5 h before staining. Cells were washed once with PBS and stained with cell surface markers for 15 min at room temperature. Intracellular markers were stained for 1 h at room temperature.

### Treg cells function analysis

Mice were euthanized at indicated timepoints through carbon dioxide inhalation followed by cervical dislocation. Tumor draining lymph nodes or right inguinal lymph nodes were collected for further analyzed. Single-cell suspensions of lymphocytes isolated from the lymph nodes were prepared by grinding organs through a pestle. Cells were washed once with PBS and stained with cell surface markers for 15 min at room temperature. Cells were fixed and permeabilized for intracellular antigens assay using a Foxp3/transcription factor staining buffer set (eBiosciences). Intracellular markers were stained for 1 h at room temperature. To analyze LAP and IL-10 expression by Treg cells, cells were stimulated with precoated anti-CD3 (1 μg/ml, Biolegend), anti-CD28 (0.5 μg/mL, eBioscience) and mIL2 (0.2 μg /mL, PeproTech) for 24 h. Brefeldin A (eBioscience) was added 5 h before staining.

### Skin T cells analysis

The skin tissue around the tumor injection site was removed after hair removal. Moreover, 20 mg of skin tissue was chopped into small pieces and incubated in collagenase type I (sigma, 5 mg/ml) and DNase I (sigma, 50 U/ml) for 45 min. The skin pieces were meshed and filtered through a 40 µm strainer. The single-cell suspension was prepared for T cells staining. Precision count beads (Biolegend) were added before acquisition to calculate absolute cells count.

### Antibodies for flow cytometry detection

Fluorochrome-tagged antibodies used were: anti-mouse CD45 (30-F11), CD3ε (17A2), CD4 (GK1.5), CD8a (53–6.7), CD39 (Duha59), CXCR3 (CXCR3-173), IL-10 (JES5-16E3), PD-1 (RMP1-30), CTLA-4 (UC10-4B9), TIGIT (1G9), LAP (TW7-16B4) from Biolegend; anti-mouse IFNγ (XMG1.2), Ki67 (16A8) from BD Biosciences; anti-mouse TNFα (MP6-XT22), Granzyme B (NGZB), Foxp3 (FJK-16 s), and fixable viability dye eFluor 780 from eBiosciences.All stained samples were detected on LSRFortessa (BD Biosciences).

### Depletion of immune cells

To deplete target cells, anti-mouse CD3ε F(ab′)_2_ fragment (145-2C11 f(ab′)_2_ fragment), CD4 (GK1.5), and CD8α (2.43), Ly6G (1A8) mAbs (BioXcell) were injected intraperitoneally (i.p.) once a week from day -1 before CMS5 cells rechallenge at a dose of 200 μg . To deplete NK cells, anti-mouse/rat asialo GM1 polyclonal antibodies (Cederlane labs) were injected i.p. 800 μg per dosage and every 4 days after the first treatment on day -1 before CMS5 cells rechallenge.

Tregs were depleted 5 days post tumor inoculation (DPI) by injecting i.p. 500 ng diphtheria toxin (Sigma). FTY720 (Sigma) 1 mg/kg was injected i.p. every day since day -1 before CMS5 cells rechallenge.

### Cytokines detection by ELISA

Samples were collected at the indicated time to detect cytokines released into peripheral blood, skin, and in vitro coculture suspension. Peripheral blood samples were centrifuged to separate serum, and 40 mg skin tissues were homogenized and centrifuged to collect suspension. Cytokine concentration in these samples was detected by a pre-coated ELISA kit (Multi Sciences) according to the manufacturer’s protocols.

### Statistical analysis

Results were presented as mean $\pm$ SEM. Data were analyzed using unpaired Student’s t-test between two groups and two-way ANOVA followed by multiple comparisons for multi-factor analysis of variance. All statistical analyses were performed on GraphPad Prism. *P* values less than 0.05 were considered significant.

## Supplementary Information

Supplementary Figures.

## Data Availability

The datasets generated during and/or analyzed during the current study are available from the corresponding author on reasonable request.
