# Supplementary material for: Transient regulatory-T-cell interruption promotes skin-resident memory T cells mediated tumor protection
Source: Sci Rep. 2023 Jul 5;13:10898. doi: 10.1038/s41598-023-36884-w (PMC10322828; doi:10.1038/s41598-023-36884-w)
Supplement: Supplementary file 1 — Supplementary Figures. [file 41598_2023_36884_MOESM1_ESM.docx]

Transient Regulatory-T-Cell Interruption Promotes Skin-Resident Memory T Cells Mediated Tumor Protection

Shushu Zhao^1^^, Shuting Wu^1^, Sheng Jiang^1^, Xiaoyu Zhou^1†^, Gan Zhao^1‡^, Bin Wang^1,2,3,4^*

^1^Key Laboratory of Medical Molecular Virology of the Ministry of Health and Ministry of Education, School of Basic Medical Sciences, Fudan University, Shanghai 200032, China

^2^Shanghai Institute of Infectious Disease and Biosecurity, Fudan University, Shanghai 200032, China

^3^National Clinical Research Center for Aging and Medicine, Huashan Hospital, Fudan University, Shanghai 200032, China

^4^Lead Contact

^^^Present address: The Wistar Institute, Philadelphia, PA 19104, USA

^†^Present address: Department of Genetics, Yale University School of Medicine, New Haven, CT 06520, USA.

^‡^Present address: Advaccine Biopharmaceutics (Suzhou) Co. LTD, Suzhou 215000, China.

*Correspondence: [bwang3@fudan.edu.cn](mailto:bwang3@fudan.edu.cn)

**SUPPLEMENTARY FIGURES**

**Figure S1. Treg depletion induced long-term tumor protection**

(**A**) Treg depletion effect by DT treatment. Peripheral blood samples from DEREG mice bearing CMS5 or not were collected at indicated time points and analyzed by flow cytometry (n = 6). **(B)** The tumor protection effect is provided by tumor excision. CMS5 tumors were excised 15 days post-tumor inoculation. Mice with tumor excision were rechallenged with CMS5 tumors s.c. 30 days post-first tumor inoculation. With mice getting a surgical incision as sham control, tumor development was monitored (n = 6). Two-way ANOVA followed by multiple comparisons for tumor growth curve analysis was used. Error bars represent mean ± SEM.

**Figure S2. T cell responses promoted rechallenged tumor rejection.**

(**A**) The gating strategy of T cells function analysis by flow cytometry and the representative results of PMA and ionomycin stimulation. (**B**) The representative flow cytometry results of CD8^+^T cells production of TNF$\alpha$ and IFN$\gamma$. (**C**) Schematic illustration of serum cytokines detection. Peripheral blood samples were collected at indicated time points. Serum cytokines IFN$\gamma$ **(D)** and TNF$\alpha$ were analyzed by the ELISA method (n = 4-6). **(E)** Gating strategy of Treg cells analysis. **(F)** Representative plots of Ki67 and PD-1 expression by Tregs from tumor draining lymph nodes. Unpaired Student’s t-test was used. ** p < 0.01, * p < 0.05. Error bars represent mean ± SEM.

**Figure S3. The rechallenged tumors were rejected even with circulating immune cell depletion.**

**(A)** Antibody-mediated CD4^+^T or CD8^+^T cells depletion effect analysis (n = 7). (**B**) Effect analysis of antibody-mediated depletion of NK or neutrophil cells (n = 6). **(C)** Analysis of peripheral immune cell reduction by FTY720 treatment (n = 5). An unpaired Student’s t-test was used. **** p < 0.0001, *** p < 0.001, ** p < 0.01, * p < 0.05. Error bars represent mean ± SEM.

**Figure S4. sT_RM_ cells distribution in the skin.**

(**A-B**) Skin T cells and sT_RM_ cells analysis with FTY720 treatment. The DEREG mice with primary CMS5 rejection were rechallenged with CMS5 tumors and treated with FTY720. T cells in tumor-adjacent skin tissues were analyzed by flow cytometry (n = 5). An unpaired Student’s t-test was used. *** p < 0.001, ** p < 0.01, * p < 0.05. Error bars represent mean ± SEM.

**Figure S5. sT_RM_ deficiency resulted in rechallenged tumor progression.**

(**A**) Experimental description of CMS5 tumor rechallenge without peripheral T cells. Tumor growth was shown in **(B)** (n = 5). (**C**) T cells depletion effect by i.p. anti-CD3ε F(ab2)’ fragment antibodies injection (n = 5). **(D)** The effect of T cells depletion caused by i.p and i.d. anti-CD3ε F(ab2)’ fragment antibodies injection was detected in multiple organs. **(E)** Analysis of CD3^+^T cell number in the skin with an antibody treatment. An unpaired Student’s t-test was used. *** p < 0.001, ** p < 0.01, * p < 0.05. Error bars represent mean ± SEM.
